# Safety and effectiveness of direct oral anticoagulants in patients with nephrotic syndrome: a report of 21 cases

**DOI:** 10.1186/s12882-022-02929-x

**Published:** 2022-09-05

**Authors:** Sarah Kelddal, Anne-Mette Hvas, Erik Lerkevang Grove, Henrik Birn

**Affiliations:** 1 Department of Renal Medicine, Aarhus University Hospital, Aarhus, Denmark; 2 Department of Biomedicine, Aarhus University Health, Aarhus, Denmark; 3 Faculty of Health, Aarhus University, Aarhus, Denmark; 4 Department of Clinical Medicine, Aarhus University Health, Aarhus, Denmark; 5 Department of Cardiology, Aarhus University Hospital, Aarhus, Denmark

**Keywords:** Nephrotic syndrome, Glomerulonephritis, Hypoalbuminemia, Proteinuria, Thromboembolic events, Prophylactic anticoagulation, Direct oral anticoagulants

## Abstract

**Background:**

Nephrotic syndrome (NS) is associated with increased risk of thromboembolic events (TE) adding to the morbidity and mortality. International guidelines recommend prophylactic anticoagulation in patients with NS and high risk of TE, but no studies have identified the optimal type of anticoagulation in NS. We aimed to assess the effectiveness and safety of direct oral anticoagulant (DOAC) by analyzing the thromboembolic and bleeding events in NS patients prescribed DOAC as primary prophylaxis to prevent TE or as treatment for TE occurring in relation to NS.

**Methods:**

We performed a single-center, retrospective study including patients with NS, a plasma albumin less than 25 g/L and prophylactic anticoagulation treatment with DOAC at the Department of Renal Medicine at Aarhus University Hospital, Denmark from July 2016 to June 2021. Patients treated with DOAC as thromboprophylaxis for other indications than NS were excluded. Baseline characteristics and outcomes, including TE, bleeding and other adverse effects associated with DOAC were obtained from medical records.

**Results:**

We identified 268 patients treated with DOAC of which 21 patients with NS were included in the study. Nineteen patients were prescribed DOAC as thromboprophylaxis and two patients received DOAC due to previous TE, which was considered associated with the NS. The type of DOAC prescribed was apixaban (*n* = 10) and rivaroxaban (*n* = 11). No patients experienced TE during DOAC treatment, while five patients had a minor bleeding episode. Patients who experienced bleeding episodes were older (median 62 vs 51 years), more often female (80%) and had been on DOAC for a longer period (204 days vs 47 days). Neither the HAS-BLED score nor GN-risk-score predicted the risk of minor bleedings in this population.

**Conclusions:**

In this case series, no new TE and only minor bleeding complications were observed among adult NS patients treated with DOAC.

## Introduction

Nephrotic syndrome (NS) is characterized by excessive proteinuria (> 3.5 g/day), hypoalbuminemia, and edema [1]. It is associated with a variety of different glomerular pathologies, including membranous glomerulonephritis, focal and segmental glomerulosclerosis, minimal change disease, and IgA nephropathy [2]. The glomerular filtration barrier defect in NS leads to urinary loss of natural anticoagulants and other plasma proteins, which stimulates hepatic protein synthesis, including the synthesis of coagulation-related proteins [1, 3–5]. These changes cause a hypercoagulable state with an up to 25% increased risk of TE that may be even further increased in patients with membranous glomerulonephritis [5, 6]. Deep vein thrombosis and pulmonary embolism has overall been associated with a 6% and 12% 30-day mortality, respectively [7], but have never been evaluated in NS patients.

The hypercoagulability in NS is not fully understood but has been ascribed to at least three different mechanisms. First, elevated thromboxane A2 may increase platelet activation and aggregation [1, 4]. Second, urinary loss of natural anticoagulants such as antithrombin and protein S combined with increased hepatic synthesis of fibrinogen and coagulation factor V and VIII results in a prothrombotic state [1, 4, 5]. Finally, decreased plasmin levels due to urinary loss, in combination with increased plasminogen activator inhibitor-1 levels, result in decreased fibrinolytic activity [4]. Given this lack of knowledge, international guidelines recommend prophylactic anticoagulation for patients with severe NS defined by the underlying condition and serum-albumin. Thus, prophylactic anticoagulation is recommended to patient with plasma-albumin below 24 g/L if membranous nephropathy and below 20 g/L in other conditions [8, 9].

Despite international guidelines recommending pharmacological thromboprophylaxis in patients with NS and high risk of TE [9], it is currently not known whether or which type of prophylactic treatment are of benefit to these patients [10]. Two trials have examined the effect of low-molecular-weight heparin (LMWH) in patients with NS suggesting that LMWH was safe and effective in preventing TE [11, 12], but the studies did not include a control group. One uncontrolled, retrospective study compared the use of LMWH and warfarin as thromboprophylaxis in NS with untreated NS patients. No episodes of TE were identified in NS patients receiving prophylactic anticoagulation, however major bleeding episodes occurred in patients on concomitant aspirin [13]. DOAC represents a newer form of anticoagulation proven superior to LMWH and warfarin in preventing TE in other conditions, such as atrial fibrillation and following variety of orthopedic surgery procedures. Notably, the use of DOAC has been associated with fewer bleeding episodes and lower mortality [14–16]. So far, the use of DOAC as thromboprophylaxis in NS has been reported in only two case reports, with mixed results as one suggested treatment failure and the other indicated a favorable response [17, 18]. In a pilot study, 16 patients with confirmed venous TE and NS were randomized to receive rivaroxaban or LMWH as treatment for TE showing no difference in the primary outcome measure, which was the absence of major bleeding episodes [19].

The purpose of this study was to evaluate the effectiveness and safety of DOAC in NS by analysing the thromboembolic and bleeding events in adult patients with NS receiving DOAC to prevent or treat TE.

## Materials and methods

### Design and study population

The study was a single centre, case series of incident or relapsing adult NS patients receiving DOAC as primary thromboprophylaxis or as treatment for TE in relation to NS from July 2016 to June 2021 at the Department of Renal Medicine, Aarhus University Hospital, Denmark. As per local guidelines, patients with established membranous glomerulonephritis should receive primary thromboprophylaxis if the plasma-albumin concentration was less than 25 g/L, whereas other patients with NS should receive thromboprophylaxis if the plasma-albumin concentration was less than 20 g/L. DOAC was prescribed per discretion of the treating physician. All patients receiving DOAC at the Department of Renal Medicine during the defined period were identified through an electronic search of all medical records. Patients meeting the inclusion criteria, most notable having NS, and no exclusion criteria were identified by a manual review of medical records. The inclusion criteria were: 1) a urinary protein excretion > 3.5 g/day, a urinary albumin excretion > 2.2 g/day and/or a urinary albumin/creatinine ratio > 2200 mg/g in combination with 2) a plasma albumin concentration < 25 g/L, 3) age > 18 years and 4) DOAC as primary thromboprophylaxis in NS or treatment for TE. Exclusion criteria were the prescription of DOAC for other indications (e.g., atrial fibrillation) and/or renal replacement therapy. The access to patient files as part of the study was approved by the Danish Data Protection Agency (reference number 1–16-02–406-21) and patient consent was not required under Danish law. All study procedures adhered to the Declaration of Helsinki.

### Electronic medical files in the Danish healthcare system

The Danish health care system is tax financed providing free health care visits and admission to all citizens. Aarhus University Hospital is the major tertiary hospital within the Central Denmark Region with no private nephrology consults available within this region. The electronic medical file contains the patient's medical current and previous history, medical prescriptions, laboratory tests, imaging, relating to both outpatient visits and admissions at public for all hospitals within the region. In addition, the electronic records provide information on all laboratory and imaging analysis prescribed by general practitioners but does not include records from these. It is, however, unlikely that any TE or significant bleeding episode would be diagnosed or treated outside a public hospital.

### Outcome

Baseline characteristics included: demographic information, plasma albumin, blood haemoglobin, platelet count, plasma creatinine, urine albumin, kidney biopsy histological diagnosis, and the type and duration of DOAC treatment. The biochemical analyses were performed using automated, standardized clinical assays at the local laboratory. Patients were followed from the start date of DOAC treatment to the end of the treatment period or end of study period. Outcomes were any TE or bleeding episodes recorded in medical records and were based on clinical symptoms or findings that would lead to relevant examinations as judged by the treating physician. Asymptomatic patients were not regularly screened for TE by ultrasound or similar. Using the International Society on Thrombosis (ISTH) bleeding scale, bleedings were classified as major or clinically relevant non-major bleeding, referred to as minor bleeding throughout this paper [20]. The CHA_2_DS_2_-VASc score was calculated based on the presence or absence of congestive heart failure, hypertension, diabetes mellitus, prior stroke or cerebral ischemia, vascular disease, age and gender, while the HAS-BLED score calculation was based on the presence or absence of hypertension, abnormal kidney and/or liver function, prior major bleeding, age, previous stroke, Labile INR, drugs and/or alcohol abuse [21, 22]. The GN-risk-score was calculated using an online tool (https://www.med.unc.edu/gntools/bleedrisk.html), which estimate the ATRIA Bleeding Risk Category (Low Risk, Intermediate Risk or High Risk) in patients with membranous nephropathy. The GN-risk-score is used to estimate the risk of bleeding versus the benefit of anticoagulation based on age, gender, race, kidney function, serum albumin, haemoglobin level and history of haemorrhage and hypertension [23].

### Data analysis

Categorical variables are presented as numbers and frequencies for baseline demographics and outcome variables, while continuous variable values are presented as median and interquartile range (IQR). Statistical analyses were performed using STATA software version 14.0 (StataCorp, College Station, TX, USA).

## Results

**Fig. 1:**
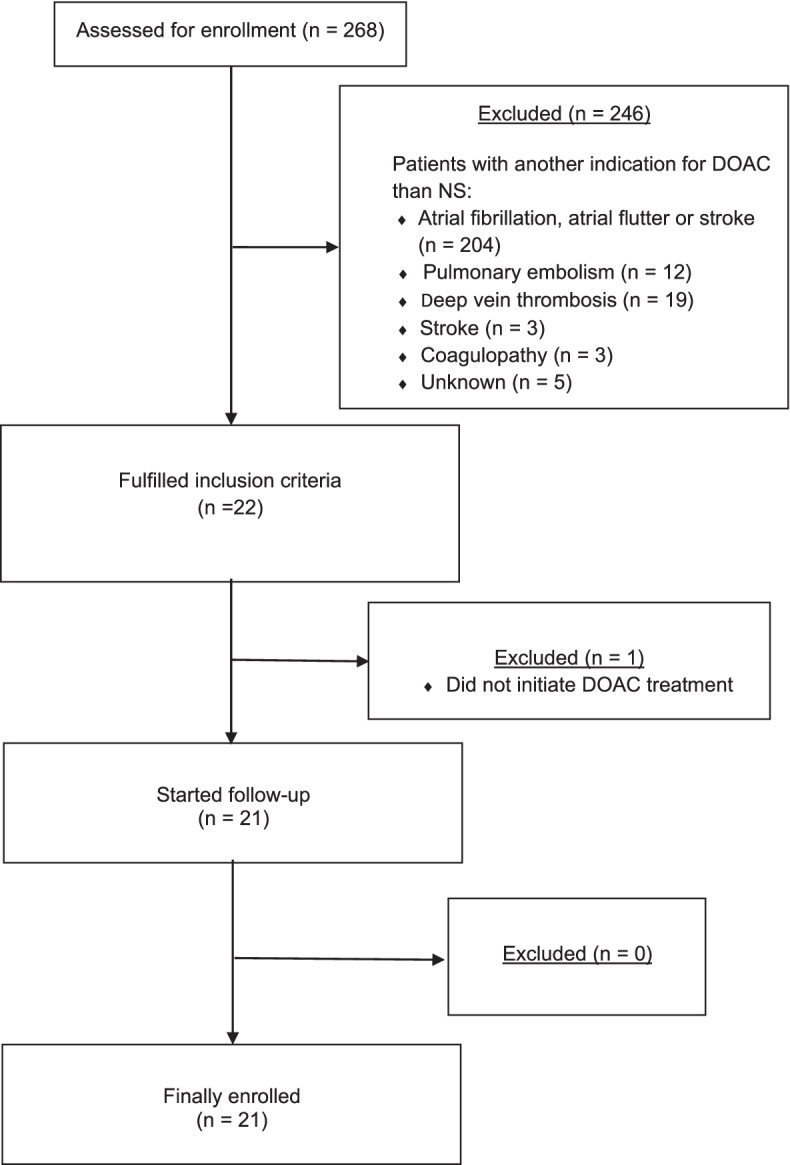
Flow-chart of patient inclusion

A total of 268 patients were screened of which 246 patients did not meet the inclusion criteria as they were treated with DOAC for other indications than primary prophylaxis in NS, mostly atrial fibrillation, atrial flutter, or stroke. One patient with NS was prescribed DOAC but did not initiate the treatment (Fig. 1). Thus, the study enrolled 21 patients of which two had a TE leading to the initiation of DOAC, while 19 patients were prescribed DOAC as primary thromboprophylaxis. During the inclusion period, one patient experienced two episodes of NS, both of which were treated with DOAC as thromboprophylaxis. Both episodes were included in the description of type of DOAC, duration of DOAC and cause of DOAC cessation.

Baseline characteristics of all patients including the CHA_2_DS_2_-VASc score, HAS-BLED score and GN-risk-score, are summarized in Table 1.

**Table 1:** Baseline data in nephrotic patients treated with direct oral anticoagulants

Baseline characteristics	All patients (*N* = 21)	Patients with no bleeding episodes (*N* = 16)	Patients with bleeding episodes (*N* = 5)
Age – years (median, (IQR))	57 (33–62)	51 (31–61)	62 (61–62)
Female sex – n (%)	11 (52)	7 (44)	4 (80)
Preexisting coagulation disorder – n (%)	1 (5)	0 (0)	1 (20)
Autoimmune disease – n (%)	2 (10)	1 (6)	1 (20)
Diabetes – n (%)	1 (5)	1 (6)	0 (0)
Liver disease	0 (0)	0 (0)	0 (0)
Atrial fibrillation	0 (0)	0 (0)	0 (0)
Cancer disease	0 (0)	0 (0)	0 (0)
Preceding thromboembolic event – n (%)	2 (10)	1 (6)	1 (20)
Histological diagnosis – n (%)			
MGN	7 (33)	5 (31)	2 (40)
FSGS	2 (10)	1 (6)	1 (20)
MCD	7 (33)	6 (38)	1 (20)
IgAN	1 (5)	0 (0)	1 (20)
Amyloidosis	4 (19)	4 (25)	0 (0)
Anticoagulation just prior to DOAC – n (%)			
LMWH	1 (5)	1 (6)	0 (0)
Concomitant anti-platelet treatment – n (%)	1 (5)	0 (0)	1 (20)
CHA_2_DS_2_-VASc score, distribution – n (%)			
0	10 (48)	10 (63)	0 (0)
1	8 (38)	4 (25)	4 (80)
2	3 (14)	2 (13)	1 (20)
HAS-BLED score, distribution – n (%)			
0	16 (76)	13 (81)	3 (60)
1	4 (19)	3 (19)	1 (20)
2	1 (5)	0 (0)	1 (20)
GN-risk-score – n (%)			
Low risk	19 (90)	15 (94)	4 (80)
Intermediate risk	1 (5)	0 (0)	1 (20)
High risk	1 (5)	1 (6)	0 (0)

*MGN* Membranous glomerulonephritis, *FSGS* Focal and segmental glomerulosclerosis, *MCD* Minimal change disease, *IgAN* IgA nephropathy, *IQR* Interquartile range, *LMWH* Low-molecular-weight heparin, *DOAC* Direct oral anticoagulants

Fifty two percent of patients were female; the median age was 57 years; the HAS-BLED score ranged from 0 to 2; and the GN-risk-score range from low risk to high risk. The majority of patients were diagnosed with membranous glomerulonephritis (33%) or minimal change disease (33%). Fifteen patients were treated with corticosteroids at some point during the study period. The samples listed in Table 2 were collected ± 14 days of the first dose of DOAC. The eGFR, plasma albumin, and urine albumin creatinine ratio recorded at termination of DOAC were collected as close as possible to the DOAC discontinuation date ranging from 16 days before the DOAC termination day to 26 days after. The mean plasma albumin concentration at initiation was 16 g/L suggesting that DOAC was prescribed appropriately in accordance with current recommendations. As expected, since low plasma-albumin was one of the main indications for primary thromboprophylaxis, plasma-albumin was higher when finishing DOAC compared to the time of initiation. Similarly, the urine albumin creatinine ratio was lower at the end of DOAC treatment (Table 2).

**Table 2:** Biochemical characteristics in nephrotic patients treated with direct oral anticoagulant

Biochemical characteristics	All patients (*N* = 21)	Patients with no bleeding episodes (*N* = 16)	Patients with bleeding episodes (*N* = 5)
**eGFR (mL/min/1.73m**^**2**^**)**			
DOAC start (median, (IQR))	85 (78–97)	83 (76–95)	96 (83–105)
DOAC end (median, (IQR))	69 (49–101)	71 (38–107)	69 (60–74)
**Plasma albumin (g/L)**			
DOAC start (median, (IQR))	16 (14–18)	15 (14–18)	17 (16–18)
DOAC end (median, (IQR))	25 (21–27)	25 (21–27)	25 (24–36)
**Urine albumin creatinine ratio (mg/g)**			
DOAC start (median, (IQR))	5268 (3219–7799)	5484 (3960–7939)	2236 (1985–6949)
DOAC end (median, (IQR))	3104 (1882–4739)	3545 (1912–4151)	2080 (26–4722)
**Hemoglobin (mmol/L, median (IQR))**	8.5 (7.9–9.0)	8.6 (7.9–8.9)	8.0 (7.9–10.1)
**Platelet count (10^9/L, median (IQR))**	272 (244–338)	315 (263–338)	259 (244–260)

*eGFR* Estimated glomerular filtration rate, *IQR* Interquartile range

Ten NS episodes (45%) were treated with apixaban (8 NS episodes were treated with 5 mg twice daily and 2 NS episodes treated with 2.5 mg twice daily because of low body weight and reduced kidney function), while 12 (55%) were treated with rivaroxaban (20 mg once daily) (Table 3). The median duration of treatment was considerably shorter in patients receiving DOAC as primary thromboprophylaxis (median of 72 days) when compared to the patients with a preceding TE (median of 970 days).

**Table 3:** Anticoagulant treatment in nephrotic patients

Intervention	Nephrotic syndrome episodes (*N* = 22^a^)	No bleeding events (*N* = 17)	Bleeding events (*N* = 5)
**Type of DOAC – n (%)**			
Apixaban	10 (45)	7 (41)	3 (60)
Rivaroxaban	12 (55)	10 (59)	2 (40)
**DOAC treatment – n (%)**			
Primary prophylaxis	20 (91)	16 (94)	4 (80)
Treatment	2 (9)	1 (6)	1 (20)
**Duration of DOAC (days, median (IQR))**	103 (21–403)	47 (21–378)	204 (109–897)
Primary prophylaxis	72 (21–326)	45 (18–326)	157 (65–551)
Secondary prophylaxis	970 (478–1462)	-	-
**DOAC are stopped due to – n (%)**			
Plasma-albumin > 20 g/L	11 (50)	7 (41)	4 (80)
eGFR < 15 mL/min/1.73m^2^	3 (14)	3 (18)	0 (0)
Ongoing at end of study	6 (27)	5 (29)	1 (20)
Other	2 (9)	2 (12)	0 (0)

*IQR* Interquartile range

^a^ One patient experienced relapse of nephrotic syndrome during the follow up period

No TE was recorded during DOAC administration. There were no major bleeding episodes; however, five minor bleeding episodes were noted during DOAC administration occurring at a median of 204 days [109–897] after commencement of treatment. No differences in histological diagnosis, type of DOAC, HAS-BLED score or GN-risk-score were observed when compared to patients without bleeding.

The minor bleeding episodes included macroscopic hematuria, cutaneous hematoma, abnormal menorrhagia, and subconjunctival bleeding. All recorded bleeding episodes were in patients with a HAS BLED score of 0 (*n* = 3) or 1 (*n* = 2) and GN-risk-score of low risk (*n* = 4) and intermediate risk (*n* = 1). One patient with a minor bleeding episode received concomitant antiplatelet treatment.

## Discussion

In this study, no episodes of TE were observed in patients with NS when treated with DOAC for a mean of 72 days. The vast majority (*n* = 19, 90%) of patients were prescribed DOAC as primary thromboprophylaxis, while 2 (10%) patients was commenced on DOAC after a TE associated with NS. In most cases, the discontinuation of DOAC was due to an increase in plasma albumin or a decline in eGFR below 15 mL/min/1.73m2. The use of DOAC was not associated with any major bleedings, whereas minor bleeding episodes were identified in 23% of patients. Neither the HAS-BLED score nor the GN-risk-score predicted the risk of minor bleedings. Corticosteroid treatment is known to increase the risk of thromboembolic events as well as the risk of gastrointestinal bleeding in the general population [24, 25]. Fifteen patients were prescribed corticosteroids during the study, two of which had a non-gastrointestinal, minor bleeding episode while on treatment.

Previous research has shown that the risk of TE in NS patients varies significantly depending on the degree of the diagnostic screening and the underlying disease, with the highest risk in patients with membranous glomerulonephritis [6, 26]. In a cohort study including 206 NS patients not receiving prophylactic anticoagulation the overall incidence of TE was 6.8%. These patients had a serum albumin level below 19 g/L with different types of pathological leading to NS [26]. We have previously reported an incidence of TE of 12% in a cohort of 35 NS patients also not receiving thromboprophylaxis and with a plasma albumin ranging from 11–29 [13]. In the cohort, on which the GN-risk-score is based, the frequency of TE in NS due to membranous glomerulonephritis and a serum albumin < 20 g/L was 11% [8]. Based on these data, we estimate that the expected incidence of TE in this case series would be between 0.2 and 1.3 patient cases if not receiving prophylactic anticoagulation.

To our knowledge, only two case reports have been published on patients receiving DOAC as thromboprophylaxis in NS. Sexton *et. al.* reported two patients with membranous glomerulonephritis and minimal change disease, respectively, prescribed apixaban as thromboprophylaxis. No TE was observed, while one patient had an episode of epistaxis, during treatment [17]. Basu et al*.* described a patient with systemic lupus erythematosus related NS and treatment failure on both VKA, LMWH and rivaroxaban [18]. It was suggested that variations in plasma coagulation factors associated with NS may have rendered anticoagulant treatment ineffective.

No randomized controlled studies have evaluated the effects of thromboprophylaxis in NS. A prospective, uncontrolled study enrolling 55 patients with NS treated with enoxaparin as thromboprophylaxis suggested that this was effective in preventing TE with no bleeding episodes [12]. Another cohort study based on medical records and including 143 NS patients receiving enoxaparin also suggested that this treatment was effective and associated with a low rate of bleeding episodes [11]. A cohort study comparing 44 NS patients receiving thromboprophylaxis (warfarin alone, warfarin with LMWH bridging, or high/low dose LMWH), with 35 NS patients not receiving prophylactic anticoagulation, reported a significant difference in the incidence of TE (0% and 12%, respectively), but also a higher frequency of bleeding events in the group receiving pharmacological thromboprophylaxis [13].

Clinical studies in other conditions have shown that DOAC is effective and safe, and this has largely replaced VKA as thromboprophylaxis in patients with atrial fibrillation and as a treatment in patients with TE [27]. Observations from clinical trials show that patients with reduced kidney function who are treated with DOAC have a higher risk of bleeding events than patients with normal renal function [28]. NS is characterized by low plasma albumin concentrations, which may cause changes in the pharmacokinetics of both apixaban and rivaroxaban, including a greater concentration of free drug as well as a greater urinary excretion, as both of these are highly protein bound [10]. These changes may potentially be associated with either lower efficacy or an increased bleeding risk. Our data indicate that DOAC is effective in preventing TE; however, given the small study size with 21 NS cases, no definite conclusions can be made. No major bleeding episodes were identified, but the incidence of minor bleeding events at 23% is higher than reported in previous studies [15, 29, 30].

The HAS-BLED and the GN-risk-score did not accurately predict minor bleeding events. The HAS-BLEED score estimates the one-year risk of major bleeding in patients with atrial fibrillation and the score has been validated in patients with atrial fibrillation on prophylactic anticoagulation, aspirin, or no treatment, and thus has not been validated in this population [21]. Since the coagulation profile of NS patients is distinct from that of patients with atrial fibrillation without renal dysfunction, this may explain why the HAS-BLED score failed to predict bleeding events. The GN-risk-score is a web-based tool, proposed by the KDIGO 2021 Clinical Practice Guideline for the Management of Glomerular Diseases, designed to assess risk of TE versus risk of bleeding events in patients with membranous glomerulonephritis treated with warfarin with an INR 2–3 dependent on plasma-albumin level [9]. It is based on previously reported incidence of TE in membranous glomerulonephritis and bleeding episodes in patients with atrial fibrillation treated with warfarin [8, 31]. The GN-risk-score did not predict bleeding events in our 21 cases, which may be explained by the variety of underlying disease in our NS population and use of DOAC rather than warfarin as prophylactic anticoagulation. Importantly, our study may underestimate the frequency of both minor bleeding episodes and subclinical TE due to the retrospective design including only clinically reported TE and bleeding episodes. The study does not address how DOAC affects the coagulation profile in NS patients, and the sample size is insufficient to conclude on the effectiveness and overall safety of DOAC use in NS patients.

Thus, although we did not observe any TE or major bleeding episodes associated with the use of DOAC in NS, additional research is required before DOAC can be recommended as a standard pharmacological thromboprophylaxis in this population.

## Data Availability

All data generated during this study are included in this article. Further enquiries can be directed to the corresponding author.

## Abbreviations

DOAC: Direct oral anticoagulants
FSGS: Focal and segmental glomerulosclerosis
IgAN: IgA nephropathy
IQR: Interquartile range
ISTH: International Society on Thrombosis and Haemostasias
LMWH: Low-molecular-weight heparin
MCD: Minimal change disease
MGN: Membranous glomerulonephritis
NS: Nephrotic syndrome
TE: Thromboembolic event
